# Are exosomes the vehicle for protein aggregate propagation in neurodegenerative diseases?

**DOI:** 10.1186/s40478-017-0467-z

**Published:** 2017-08-29

**Authors:** Yoon-Ju Lim, Seung-Jae Lee

**Affiliations:** Departments of Medicine and Biomedical Sciences, Neuroscience Research Institute, Seoul National University College of Medicine, 103 Daehak-ro, Jongro-gu, Seoul, 03080 South Korea

**Keywords:** Neurodegenerative diseases, Disease progression, Cell-to-cell transmission, Protein aggregation, Neurodegeneration

## Abstract

Abnormal protein aggregation has been implicated in neurodegenerative processes in human neurological disorders, such as Alzheimer’s disease and Parkinson’s disease. Recently, studies have established a novel concept that protein aggregates are transmitted among neuronal cells. By extension, such interneuronal aggregate transmission has been hypothesized to be the underlying mechanism for the pathological and clinical disease progression. However, the precise mechanism of the interneuronal aggregate transmission remains ill-defined. Recent reports have suggested that exosomes, a specific group of extracellular vesicles that are involved in intercellular transfer of cellular macromolecules such as proteins and RNAs, could play an important role in the aggregate transmission among neurons. Here, we review various types of extracellular vesicles and critically evaluate the evidence supporting the role of exosomes in interneuronal aggregate transmission and neurodegeneration. We also discuss the competing mechanisms other than the exosome-mediated transmission. By doing so, we aim to assess the current state of knowledge on the mechanism of interneuronal aggregate transmission and suggest the future directions of research towards understanding the mechanism.

## Introduction

Aggregation of specific proteins is the common pathological feature of neurodegenerative diseases, such as Alzheimer’s disease (AD) Parkinson’s disease (PD), and amyotrophic lateral sclerosis (ALS) [7, 22]. These protein aggregates compose different types of inclusions. In AD, amyloid β (Aβ) peptides and hyperphosphorylated tau are deposited in senile plaques and neurofibrillary tangles (NFTs), respectively [7, 22]. PD is characterized by α-synuclein aggregates in the forms of Lewy bodies and Lewy neurites [7, 22, 34]. Inclusion bodies containing aggregates of TAR DNA-binding protein 43 (TDP-43) exist in ALS patients [7, 22].

In general, neuropathological protein aggregates tend to develop at a few discrete loci in the brain and spread to other brain areas as the diseases progress. Each type of pathological aggregates exhibits its own stereotypical pattern of spreading [7, 8, 17, 22]. For example, tau inclusions in AD are first observed in the transentorhinal cortex and spread through the hippocampus to the neocortex areas [5]. On the other hand, Lewy bodies and Lewy neurites in PD may follow an ascending pattern from the lower brainstem and olfactory bulb through the midbrain and limbic system, and finally to the neocortex [6], though there have been multiple examples of cases that do not follow this pattern of progression [21]. However, whether the spreading of pathological protein aggregates itself causes neurodegeneration and disease progressions is uncertain. Nevertheless, significant correlations exist between the regional progression of aggregate pathology and the sequential development of clinical symptoms in these diseases. Therefore, we might be able to solve the mechanism of clinical disease progression by understanding the machinery underlying the aggregate spreading.

Recently, a large body of evidence has supported the involvement of cell-to-cell transmission in the aggregate spreading. Specifically, exosomes have been the subject of discussion as the vehicle for the cell-to-cell transmission. In this review, we will define the exosome in relation to other extracellular vesicles. Next, we will provide evidence for the roles of exosomes in aggregate spreading and limitations of the existing studies. Finally, we will discuss what lies ahead in comprehending the mechanism of the intercellular aggregate transmission.

### Cell-to-cell transmission of aggregated proteins

**Fig. 1:**
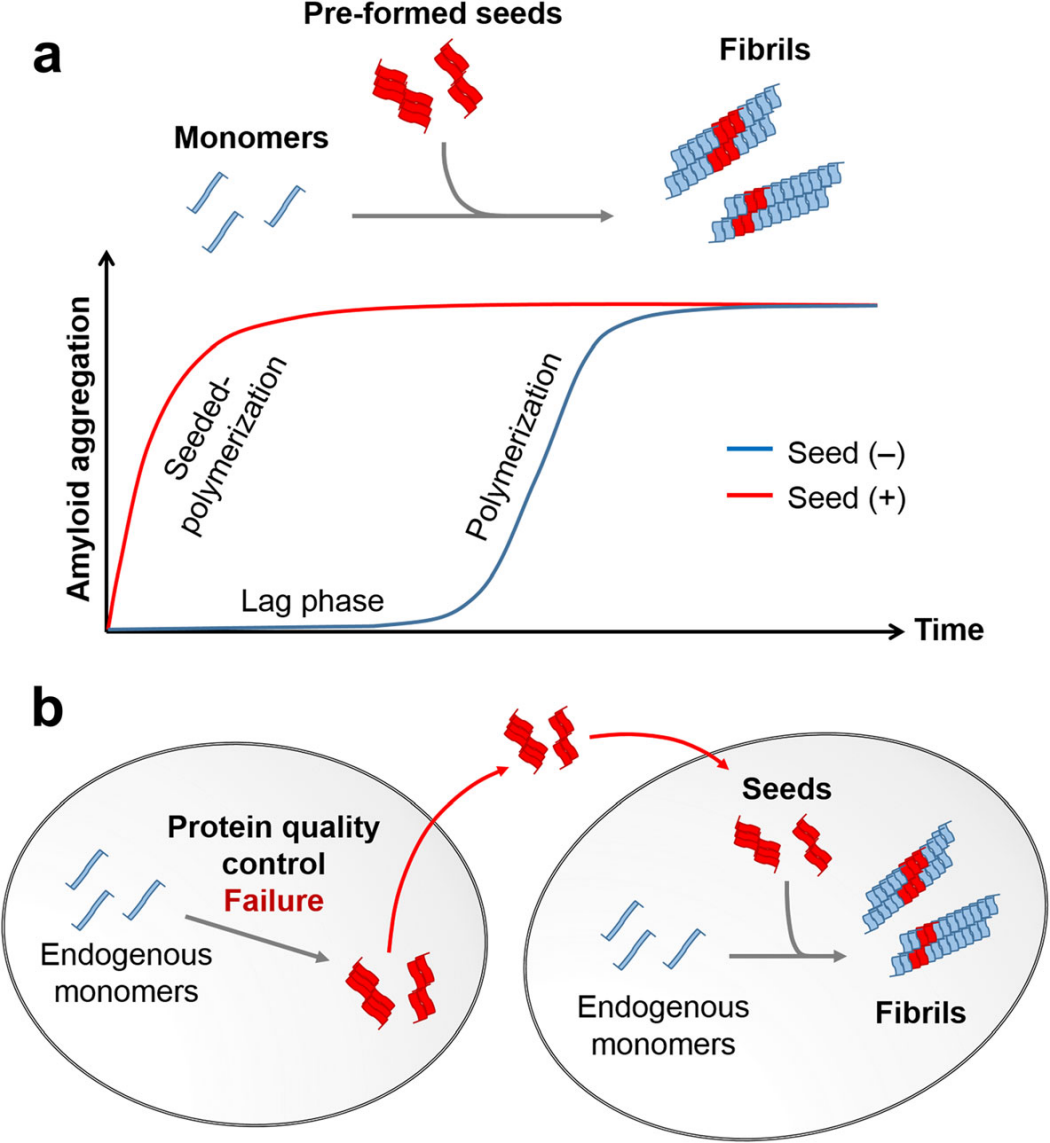
Kinetic principle of protein aggregation underlies the intercellular aggregate transmission. **a** Simplified scheme illustrating the kinetics of protein fibrillation and seeded polymerization. Addition of pre-formed fibrils drastically reduced the lag phase. **b** Illustration of seeded polymerization principle in cell-to-cell aggregate transmission. When protein aggregates are transferred from one cell to another, the transferred aggregates could act as ‘seeds’ in the recipient cells

Aggregation process is a nucleation-dependent process, which requires a nucleus formation during the lag phase [22]. When pre-formed aggregates are present, the aggregation process bypasses the lag phase and rapidly produces aggregates using the pre-formed aggregates as seeds (Fig. 1). This has been the molecular principle underlying the prion infectivity and transmissibility. The same principle can be applied to interneuronal transmission of the aggregated proteins other than prion. The seeding-dependent mechanism has not been completely proven; there are other possibilities by which the protein aggregates are propagated through the interneuronal aggregate transfer [27, 28]. Although the precise mechanism of the transmission remains ill-defined, there are ample experimental supports for interneuronal aggregate transmission in both cells and animals. However, in order for the cytoplasmic protein aggregates to propagate among the neuronal cells, they have a significant challenge: the aggregates have to be secreted from one neuron and transferred to another.

Such problem of this barrier has begun to be addressed as some experiments showed that tau, α-synuclein, and other aggregated proteins were secreted from neurons [7]. α-synuclein and tau have been shown to be secreted through an endoplasmic reticulum/Golgi-independent pathway, which are collectively referred to as unconventional secretory pathway [2, 14, 24–26, 34, 43]. Physiological functions of the secreted protein aggregates are unknown. The extracellular aggregates may regulate synaptic activity [9] and inflammatory responses [23]. However, the dichotomy between physiological and pathological functions of these extracellular aggregates remains fuzzy. Among the unconventional secretory pathways, the exosome-associated exocytosis has attracted much attention because this particular mechanism has been shown to be involved in the secretion of many disease-linked proteins, including prion, Aβ, α-synuclein and tau (see below), as well as in both physiological and pathogenic processes in human health and disease.

### Extracellular vesicles and exosomes

**Fig. 2:**
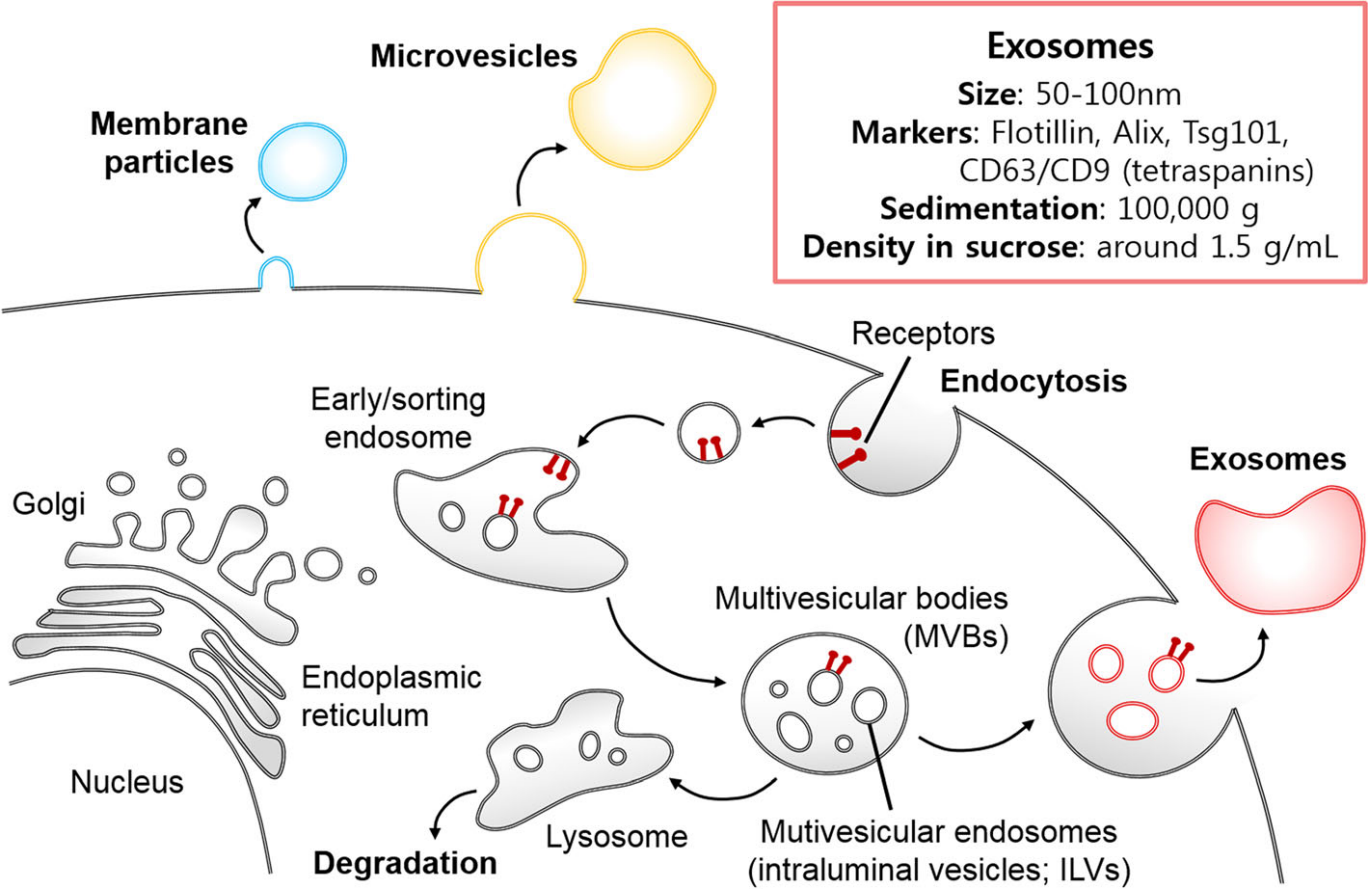
Extracellular vesicles. Only exosomes, microvesicles and membrane particles are shown here for simplification, however, more vesicle types may be present in the extracellular space. Exosomes are generated by exocytosis of MVBs, and microvesicles are formed by budding/blebbing of the plasma membrane. Membrane particles also formed by the similar mechanism as microvesicles, but the biological markers are different

Cells secrete a variety of membranous vesicles during their lives. There are many types of extracellular vesicles: apoptotic bodies, microvesicles/membrane particles, exosomes, etc. (Fig. 2). Each type of these vesicles has its own size, marker proteins, and different secretion pathway. Microvesicles are large (>100 nm diameter) membranous vesicles produced by shedding/budding/blebbing from the plasma membrane of various cell types [19, 31, 36, 38, 39]. These vesicles have irregular shapes and their biomarkers, such as integrins, selectins, and CD40 [31]. Membrane particles are also originated from the plasma membrane and are round-shaped vesicles in 50-80 nm diameter. CD133 (prominin-1) is their biomarker, not CD63 [30]. On the other hand, exosomes are intraluminal vesicles (ILVs) within the multivesicular bodies (MVBs) secreted when MVBs are fused with the plasma membrane. They are 50-100 nm in diameter identified by methods such as electron microscopy (EM) and nanoparticle tracing analysis (NTA). Although there are descriptions on exosomes and other extracellular vesicles, currently there is no consensus criteria to define different types of EVs [32, 47]. Extracellular vesicles (particularly microvesicles and exosomes) often overlap in size and share some surface markers (e.g., tetraspanins CD9, CD63, CD81, etc.) [19, 29, 31, 36, 38, 39]. The cup-shaped morphology of exosomes is known to be an artifact of TEM fixation [41]. More studies with improved procedure for exosome preparation would be necessary for identification of specific markers for exosomes.

Exosomes are purified by the specific procedure, which involve differential ultracentrifugation with cell culture supernatant followed by a rate-zonal centrifugation (Table 1) [41]. Exosomes are located at the 1.10–1.20 g/mL density fraction [19, 31, 38, 39]. Although this procedure has been widely used, some contamination of serum components could not be avoided [32]. There are alternative procedures available, including size exclusion, immunoaffinity isolation, and polymeric precipitation [45]. However, the benefits of these procedures over the conventional centrifugation procedure has not been established yet. Exosomes and other extracellular vesicles may function as vehicles for transportation of macromolecules from one cell to another. Exosomes contain not only proteins but also RNAs and DNAs. Transfer of these macromolecules may play roles in several biological processes, such as innate and acquired immunity and angiogenesis, as well as in human diseases, such as neurodegenerative diseases and carcinogenesis and metastasis [47].

**Table 1:** Procedure of exosome preparation [41]

***1. Differential ultracentrifugation***
Culture supernatant or fluid – at each of steps, the supernatant is used for following step.
⇒ 300 x g, 10 min (→ Pellet: cells) ⇒ 2000 x g, 10 min (→ Pellet: dead cells) ⇒ 10,000 x g, 30 min (→ Pellet: cell debris) ⇒ *Final* 100,000 x g, 70 min → Pellet: *exosomes* + contaminating proteins ⇒ Wash in PBS 100,000 x g, 70 min → Pellet: correspond to *exosomes*
***2. Rate-zonal centrifugation***
- Load exosome preparation at the gradients (0.25 - 2 M sucrose) and centrifuge overnight (≥14 h) at 210,000 x g, 4 °C. - With a micropipettor, collect eleven 1-mL fractions, from top to bottom.

### Literature arguing the role of exosomes in aggregate transmission

Recent studies suggest that exosomes play important roles in interneuronal transmission of pathogenic proteins and neurodegeneration. The first example was provided in a prion model. Both cellular prion (PrPc) and scrapie form of prion (PrPsc) proteins were released into the extracellular space in association with exosomes [16]. The exosome-associated PrPsc was infectious [16]. Later, it was shown that some Aβ peptides generated from the β- and γ-cleavage of amyloid precursor protein (APP) in endosomes were routed to MVBs and secreted by the exosomal pathway [35]. Furthermore, exosomal proteins were found in the amyloid plaques of AD patient brains [35]. Injection of the exosome preparations into 5xFAD mouse brains promoted aggregation of Aβ1–42 [13]. Inhibition of exosome-associated exocytosis by intraperitoneal injections of GW4869, an inhibitor of neutral sphingomyelinase-2 (nSMase2; a ceramide synthesis regulator which is essential for subtype of exosome biogenesis in MVBs) reduced the Aβ1–42 plaque load in vivo [13]. These suggest that exosomes have their role in AD pathology, triggering aggregation and deposition of amyloid plaques.

Tau was also shown to be secreted via the exosomal pathway [37, 44]. Exosomal tau secretion was identified in primary neurons and tau overexpressing N2a cell culture media. Exosome-associated phospho-tau (AT270-positive) was present in human CSF [37]. The exosomes with associated tau proteins were taken up by neurons and microglia, and induced tau inclusions [44]. Exosomes from CSF samples were also able to promote tau aggregation in cultured N2a cells [44]. Microglia also secrete tau via exosomes and tau propagation is reduced significantly by inhibition of exosome synthesis [4]. When microglial nSMase2 was silenced by siRNA or pharmacologically inhibited with GW4869, exosome-associated secretion of tau and propagation of tau pathology were reduced [4].

In Parkinson’s model, α-synuclein was also secreted via the exosomal pathway in a calcium-dependent manner [15]. These exosomes can be transferred to recipient cells via endocytosis [12], and the transfer was increased when lysosomes were impaired [3]. Additionally, exosome-associated α-synuclein oligomers were taken up by recipient cells more efficiently and had higher toxicity than free α-synuclein oligomers [10]. Exosomes isolated from the CSF of PD and DLB patients were shown to contain pathogenic species of α-synuclein and were able to induce oligomerization of soluble α-synuclein [40]. Injection of exosomes isolated from DLB patients into the brain led to spreading of a-synuclein aggregates [33].

Recently, another way that exosomes contribute to the disease propagation was suggested. Exosomes can accelerate in vitro oligomerization of recombinant α-synuclein monomer and increase toxicity of these proteins [18]. This study also showed that the accelerated oligomerization was partly due to the lipid contents; gangliosides of the exosomal membranes. Similarly, Aβ assembly is markedly accelerated by incubation with the exosome fraction from the PC12 cell culture media [48].

### Critiques to the exosome theory in aggregate transmission

Although there is a body of literature supporting the role of exosomes in the interneuronal aggregate propagation, there are some issues that need clarification. First, not all studies prepared exosomes in a proper way. As mentioned above, a pure exosome preparation requires both differential ultracentrifugation and rate-zonal centrifugation. Differential ultracentrifugation alone results in mixture of many different types of extracellular vesicles. Even after rate-zonal centrifugation, the exosome preparation still may contain contaminated non-exosomal vesicles, which necessitates electron microscopy analysis with immune-gold labeling [41]. As summarized in Table 2, in some studies, exosome preparations were obtained from the differential ultracentrifugation without the rate-zonal centrifugation with sucrose gradient being performed. These preparations would contain not only exosomes, but also other extracellular vesicles such as microvesicles and membrane particles.

**Table 2:** Summary of evidence supporting the exosomal transmission of pathogenic neurodegenerative disease proteins

	Size	Sedimentation	Sucrose gradient	Marker	Reference
Prion	50-90 nm	100,000 g	O	Flotillin, Tsg101	[16]
Aβ	60-100 nm	100,000 g	O	Alix, Flotillin	[35]
	Not described	110,000 g	X	Alix, Tsg101	[13]
Tau	60-100 nm	100,000 g	O	Alix	[37]
	50-100 nm	100,000 g	O	Tsg101	[4]
	40-100 nm	100,000 g	O	Alix, Flotillin	[44]
α-synuclein	50-140 nm	100,000 g	X	Alix, Flotillin	[15]
	93, 99 nm	120,000 g	X	Alix, Flotillin, LAMP1	[3]
	60-100 nm	100,000 g	X	Alix, Flotillin, CD63	[10]
	~100 nm	100,000 g	X	Flotillin	[40]

Another issue is that pathogenic proteins in association with exosomes represent only a minor part of the total secreted pathogenic proteins [14, 35]. Only a little fraction (<1%) of total secreted Aβ into the extracellular space is shown to be exosome-associated [35]. In case of α-synuclein, less than 3% of the total secreted protein seems to be associated with exosome [14]. However, low representation in quantity does not necessarily indicate insignificant functional roles. Danzer et al. [10] showed that exosome-associated α-synuclein was more toxic to cells than the exosome-free protein. However, since the study used a larger quantity of exosome-associated α-synuclein proteins than actual, the effects of actual concentration of exosome-associated α-synuclein remains unknown.

Perhaps, the most critical critique of the exosome theory of aggregate propagation may be that this theory relies mostly on phenomenology in that at least some secreted pathogenic proteins are present in the exosomes. There is little functional evidence which demonstrates the involvement of exosome-associated proteins in aggregate propagation. Genetic modifications of exosome formation and trafficking in cells and animals would be one way to resolve this issue. In light of this, the study by Hasegawa et al. [20] was alerting. In this study, when exosome biogenesis was inhibited by silencing the expression of VPS4, a protein necessary for exosome formation, secretion of α-synuclein was increased, rather than decreased. In the same study, the authors failed to detect α-synuclein proteins in exosome preparations from human CSF. However, this study did not show the effect of VPS4 silencing on intercellular a-synuclein transmission, leaving the possibility of exosome still being important for aggregate transmission.

### Alternative mechanisms

**Fig. 3:**
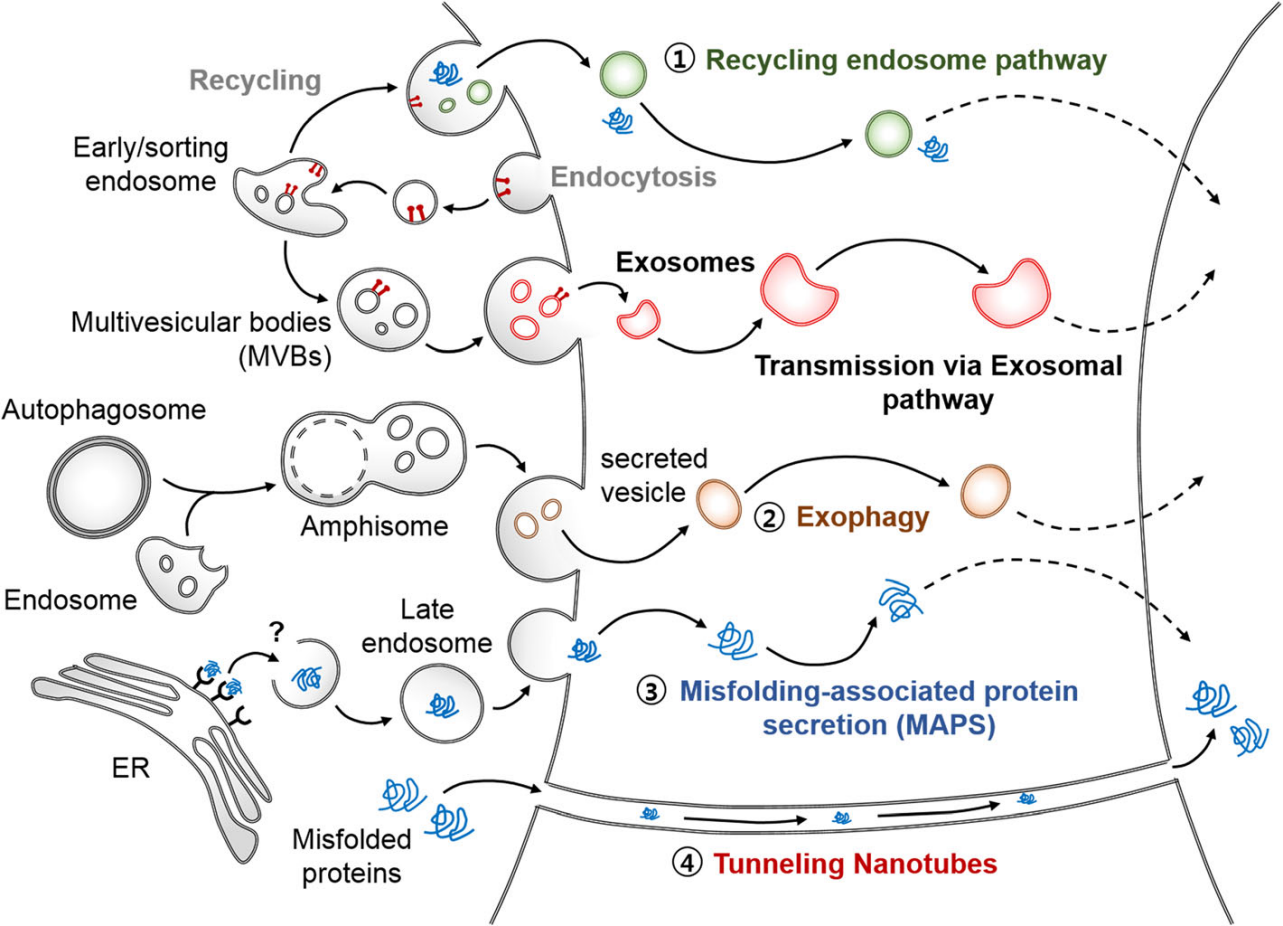
Possible mechanisms of interneuronal protein aggregate transmission alternative to the one involving exosomes

Several mechanisms other than the exosome pathway have been proposed to explain cell-to-cell aggregate transmission (Fig. 3). The endosome recycling pathway is one of those mechanisms. This pathway verified its functional importance in the cellular amyloid precursor protein trafficking and Aβ generation [46]. Another unconventional secretion that might be involved in the interneuronal aggregate transmission is exophagy, which refers to the exocytosis mediated by fusion of autophagosome/amphisome with the plasma membrane. The release of α-synuclein monomer and aggregates was mediated by exophagy, when the autophagosome-lysosome fusion was impaired [2, 14]. In PC12 cell, tubulin polymerization-promoting protein (TPPP/p25α) co-localized with α-synuclein in autophagosomes and inhibited the fusion of autophagosomes with lysosomes [14]. This led to α-synuclein secretion into the media through exophagy [14]. This study also showed that the secretion was modulated by Rab27a, a regulator of the late endosomal and amphisomal exocytosis [14].

Recently, another unconventional exocytosis mechanism has been discovered and was named as misfolding-associated protein secretion or MAPS. This mechanism was activated when the proteasomal degradation was impaired [43]. Polyubiquitylated proteins were recruited to the cytosolic surface of ER through the interaction with USP19, an ER membrane-associated deubiquitylase [26, 43]. The misfolded proteins were then deubiquitylated and packaged in the late endosome-like compartments before secreted [26, 43]. The MAPS pathway is probably a part of cellular protein quality control system, removing misfolded proteins from cells; however, this pathway might also play an important role in aggregate propagation.

Alternatively, direct intercellular transmission of pathogenic proteins through the tunneling nanotubes was recently proposed [1, 42]. Tunneling nanotubes are protrusions of actin enriched structures extended from the plasma membrane. They can be as thick as 50 to 200 nm in diameter and be as long as up to several cell diameters [11]. In this case, the cytoplasm of neurons are directly connected, which makes exocytosis of aggregates obsolete. Although the evidence for transport of proteins and organelles through tunneling nanotubes has been accumulating in culture, the presence of tunneling nanotubes in vivo, especially in the brain, has not been proven yet.

## Conclusions

A growing body of literature proposes a theory that exosomes play important roles in the cell-to-cell transmission of pathogenic protein aggregates, thereby contributing to the pathological and clinical progression of neurodegenerative diseases. The exosome theory suggests that pathogenic protein aggregates are encapsulated into ILVs in MVBs and released from cells by fusion of MVBs with the plasma membrane. Exosomes, the released ILVs containing pathogenic protein aggregates then transfer these aggregates to other cells around.

However, the current literature possesses shortcomings in proving the exosome theory. Notably, many of the studies analyzed crude exosomal preparations, which also contain many different types of extracellular vesicles other than exosomes and even free protein aggregates large enough to be sedimented at the given centrifugal forces. Quantitative analysis as to precisely how many percentages of secreted pathogenic proteins are associated with exosomes, is missing in many of these studies. More importantly, genetic and pharmacological intervention of exosome formation should be employed in investigating the role of exosomes in secretion and propagation of the pathogenic proteins.

In addition, given the confusion in the field as for the definition of exosome, perhaps, one should use the term “extracellular vesicles” rather than “exosomes”, until we understand better the components, classification, and biological functions of these vesicles.

Another important issue is related to the effects of exosome-associated pathogenic proteins on the recipient cells; how these proteins are internalized and how they induce protein aggregation and neurodegeneration in the recipient cells. It has been shown that free-forms of the pathogenic aggregated proteins can be internalized into the neuronal cells through endocytosis [7]. Therefore, the advantages of the exosome-associated protein aggregates over exosome-free forms in aggregate propagation and neurodegeneration should be addressed as well as the logical basis of these benefits. In addition, the roles of other exosomal components in aggregate propagation would be an interesting topic for the future studies.

Although many questions remain unanswered, the exosome theory is considered an attractive and partly valid explanation for the intercellular propagation of proteinopathies. Future investigation into this topic would contribute to the disclosure of the mechanism of protein aggregate propagation and the progression of neurodegenerative diseases.
